# Overcoming MITF-conferred drug resistance through dual AURKA/MAPK targeting in human melanoma cells

**DOI:** 10.1038/cddis.2015.369

**Published:** 2016-03-10

**Authors:** G Pathria, B Garg, V Borgdorff, K Garg, C Wagner, G Superti-Furga, S N Wagner

**Affiliations:** 1 Division of Immunology Allergy and Infectious Diseases (DIAID), Department of Dermatology, Medical University of Vienna, Vienna, Austria; 2 CeMM, Research Center for Molecular Medicine of the Austrian Academy of Sciences, Vienna, Austria

## Abstract

*MITF* (microphthalmia-associated transcription factor) is a frequently amplified lineage-specific oncogene in human melanoma, whose role in intrinsic drug resistance has not been systematically investigated. Utilizing chemical inhibitors for major signaling pathways/cellular processes, we witness MITF as an elicitor of intrinsic drug resistance. To search kinase(s) targets able to bypass MITF-conferred drug resistance, we employed a multi-kinase inhibitor-directed chemical proteomics-based differential affinity screen in human melanocytes carrying ectopic *MITF* overexpression. A subsequent methodical interrogation informed mitotic Ser/Thr kinase Aurora Kinase A (AURKA) as a crucial regulator of melanoma cell proliferation and migration, independent of the underlying molecular alterations, including TP53 functional status and MITF levels. Crucially, assessing the efficacy of investigational AURKA inhibitor MLN8237, we pre-emptively witness the procurement of a molecular program consistent with acquired drug resistance. This involved induction of multiple MAPK (mitogen-activated protein kinase) signaling pathway components and their downstream proliferation effectors (Cyclin D1 and c-JUN) and apoptotic regulators (MITF and Bcl-2). A concomitant AURKA/BRAF and AURKA/MEK targeting overcame MAPK signaling activation-associated resistance signature in *BRAF-* and *NRAS-*mutated melanomas, respectively, and elicited heightened anti-proliferative activity and apoptotic cell death. These findings reveal a previously unreported MAPK signaling-mediated mechanism of immediate resistance to AURKA inhibitors. These findings could bear significant implications for the application and the success of anti-AURKA approaches that have already entered phase-II clinical trials for human melanoma.

Detailed molecular investigation of human melanoma has unearthed two key oncogenic driver alterations BRAF(V600E) in ~40% and NRAS (G12D) in ~15% of melanomas.^1^ Even though the subsequent drug design effort has accomplished to provide highly specific mutated (mut)-BRAF inhibitors,^2^ albeit encouraging initial clinical responses,^2, 3^ their long-term success has invariably been jeopardized by the development of elaborate resistance mechanisms.^4, 5, 6, 7, 8, 9^ Nevertheless, the continued elucidation of resistance mechanisms that almost always restore MAPK (mitogen-activated protein kinase) signaling activity, while offering avenues for combinatorial therapeutics, re-allude to the inalienability of this signaling hub from melanoma cell biology.^4, 5, 6, 7, 8, 10^ Additionally, a significant proportion of melanomas harboring mut-*BRAF* allele remain intrinsically resistant to BRAF inhibitors. Even so, a previous report showed stromal HGF-mediated resistance to targeted BRAF inhibitors,^11^ and a recent study reported the role of stroma-mediated immediate resistance to BRAF inhibition,^12^ the general understanding of the mechanisms of intrinsic resistance has remained quite limited.

Microphthalmia-associated transcription factor (MITF) is a basic helix–loop–helix transcription factor that has critical role in melanocytic development and melanomagenesis.^13, 14^ MITF has been described as a lineage-specific oncogene in melanoma, which in collaboration with constitutively active mutated BRAF capably transforms melanocytes.^14^ MITF carries a diverse functionality and has been shown to influence a wide range of cellular phenotypes, including proliferation, apoptosis, migration and differentiation.^15^ This functional diversity has in-turn been ascribed to different MITF expression levels.^15^ Although some investigative studies in melanoma cells have also suggested a role for MITF in both intrinsic and acquired resistance to general as well as targeted therapeutics, including MAPK signaling inhibitors,^9, 14, 16, 17^ a systematic interrogation of this MITF functionality has largely gone unexplored.

In the current study, we systematically investigated the role of MITF in intrinsic drug resistance, followed by development of therapeutic strategies that thwart/bypass the liaison between BRAF(V600E) and MITF.

## Results

### MITF and intrinsic drug resistance

**Figure 1:**
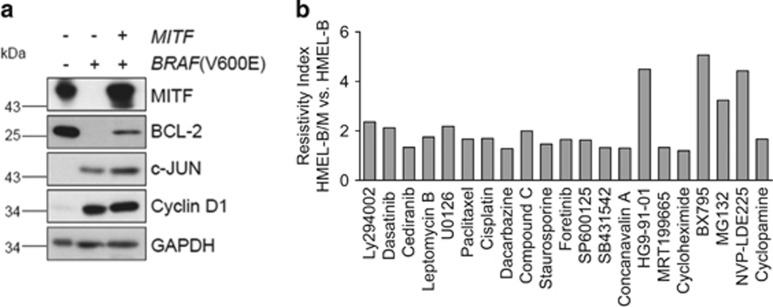
MITF confers intrinsic drug resistance in melanocytes. (**a**) Immortalized human melanocytes transformed with constitutively active *BRAF*(V600E) (HMEL-B) or a combination of *BRAF*(V600E) and *MITF(*HMEL-B/M) were analyzed for the expression of the indicated proteins by immunoblotting. (**b**) HMEL-B and HMEL-B/M cells were treated with the indicated inhibitors (Supplementary Table S1) for 24 h, followed by determination of the resistivity index (see Methods) of HMEL-B/M cells in comparison to the HMEL-B cells (*n*=3)

To explicitly understand the role of MITF in intrinsic drug resistance, we tested immortalized HMEL cells (Pmel/hTERT/CDK4(R24C)/p53DD), ectopically expressing BRAF(V600E) (referred to as HMEL-B) or BRAF(V600E)+MITF (referred to as HMEL-B/M)^14^ (Figure 1a) for their responsiveness to carefully selected targeted and general therapeutics (Supplementary Table S1). In line with the previous reports,^18, 19^ introduction of constitutively active BRAF(V600E), while triggering an induction of c-JUN and Cyclin D1 expression, markedly downregulated the expression of endogenous MITF and its target Bcl-2.^20^ However, ectopic expression of *MITF* could restore MITF levels and partially rescue its target (Bcl-2) expression (Figure 1a). The choice of this model cellular system permitted an unhindered assessment of drug–response features specifically conferred by MITF within an isogenic background. Introduction of *MITF* in HMEL-B cells greatly enhanced their resistance to a wide range of tested inhibitors (Figure 1b; Supplementary Figure S1A). In contrast, however, MAPK pathway inhibition, with the exception of MEK inhibitor U0126, utilizing multiple MAPK signaling inhibitors demonstrated equivalent or higher sensitivity of HMEL-B/M cells (Supplementary Figure S1B). With *MITF'*s qualification as a lineage-specific oncogene^14^ in human melanoma, and its tight regulation downstream of MAPK signaling^18, 21^ (Supplementary Figure S1C), the heightened sensitivity of HMEL-B/M to MAPK signaling inhibitors is not unfounded. These data corroborate similar findings demonstrating increased responsiveness of MITF^High^ melanoma cells to MAPK signaling inhibitors.^22^ Although these results may argue for MAPK inhibition as a sufficient means to overcome MITF-associated features, in light of a recent report demonstrating BRAF-i-mediated MITF induction,^17^ and the highly pervasive acquired resistance to targeted BRAF inhibition,^5, 6, 7, 8, 9^ identification of new targets able to simultaneously overcome/bypass MITF and BRAF(V600E) activities is essential.

### Integrative differential drug affinity-based proteomics

**Figure 2:**
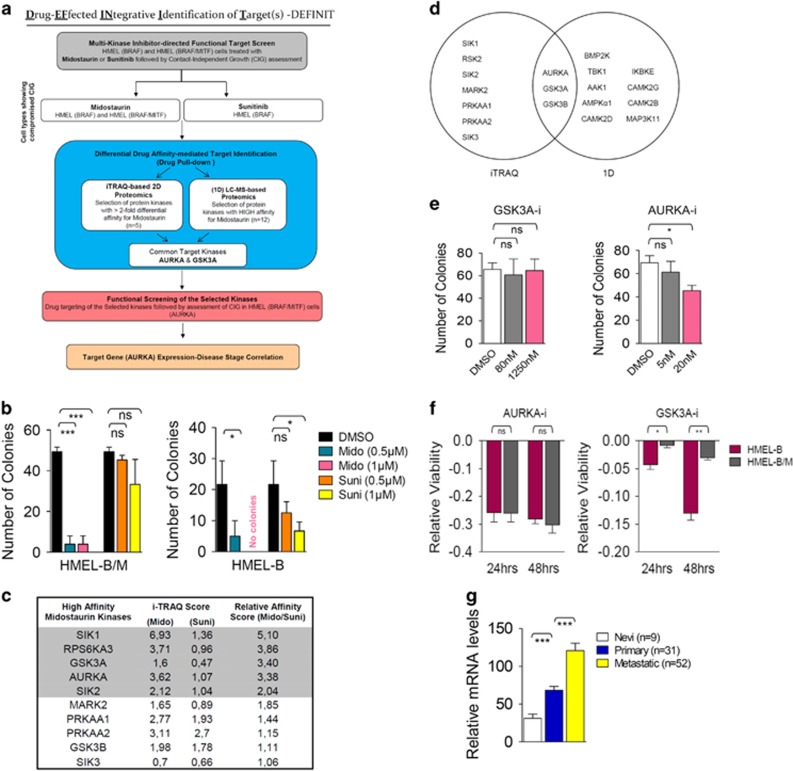
Integrative differential drug affinity-based proteomics. (**a**) Flowchart depicting the multi-step strategy (DEFINIT) for the identification of the kinase(s) targets circumventing MITF-mediated intrinsic drug resistance. (**b**) HMEL-B/M and HMEL-B cells were treated as indicated for 4 weeks in a soft agar colony formation assay followed by colony count (*n*=3). (**c**) List of top 10 kinases exhibiting higher relative affinity for midostaurin in comparison to sunitinib based on iTRAQ score. (**d**) Venn diagram depicting the exclusive and shared kinases from iTRAQ and (1D) LC–MS studies. (**e**) HMEL-B/M cells were treated as indicated for 4 weeks in a soft agar colony formation assay followed by colony count (*n*=3). (**f**) HMEL-B and HMEL-B/M cells were treated with DMSO control or AURKA inhibitor (MLN8237, 100 nM) and GSK3A inhibitor (SB415286, 5 *μ*M) for 24 or 48 h followed by assessment of relative viability (*n*=3). (**g**) Relative gene expression levels of *AURKA* in the indicated stages of melanoma. All error bars indicate ±S.D.; ns, non-significant; *P⩽0.05, **P⩽0.01, ***P⩽0.001

To identify potential target(s) for therapeutic intervention in a significant proportion of melanomas that harbor dual mut-*BRAF* and *MITF* amplification (*MITF*^Amp^),^14^ we developed a multi-step integrative target identification approach—drug-effected integrative identification of target(s) (DEFINIT). This approach, relying strongly on functional assessment, also incorporated the salient feature of ‘gene expression-disease stage correlation' (Figure 2a). With the perturbed cellular kinome being the single most unifying feature of highly heterogeneous cancers,^23^ we hypothesized an invariable association of MITF-engendered resistance with a deregulated kinome. Thus, we predicted the ability of broad-specificity multi-kinase inhibitors to overcome MITF-conferred resistance and guide the identification of specific kinase targets able to negate or bypass MITF activities. To test this premise, we investigated the effect of midostaurin, an established multi-kinase inhibitor, on the growth of HMEL-B/M cells in 3D soft agar assay, a surrogate for pro-tumorigenic phenotype *in vivo*. Notably, even at very low doses, midostaurin almost completely compromised the colony-forming capacity of both HMEL-B and HMEL-B/M cells (Figure 2b; Supplementary Figure 2A). Interestingly, however, sunitinib, carrying an overlapping target spectrum with midostaurin,^24^ while compromising the growth of HMEL-B cells, failed to show any significant effect in HMEL-B/M cells (Figure 2b; Supplementary Figure 2A). These observations argued for a set of distinct kinase targets for midostaurin and sunitinib, with the former able to additionally/differentially block the kinase(s) essential for MITF-associated drug resistance phenotype in HMEL-B/M cells. We next analyzed our previously published isobaric tag for relative and absolute quantification (iTRAQ) labeling-based quantitative proteomics data set^18^ to identify kinases that exhibit differential affinity towards midostaurin in comparison to sunitinib in HMEL-B/M cells (Supplementary Table S2). To increase the confidence level, from the 10 kinases showing differential affinity for midostaurin, we selected the ones exhibiting a relative affinity score (midostaurin/sunitinib) >2 (Figure 2c). This yielded 5 kinases—salt-inducible kinase 1 (SIK1), ribosomal S6 kinase 2 (RSK2), glycogen synthase kinase 3A (GSK3A), Aurora kinase A (AURKA) and salt-inducible kinase 2 (SIK2; Figure 2c).

Notably, a parallel assessment of the high-affinity midostaurin targets employing gel-free one-dimensional liquid chromatography–mass spectrometry ((1D) LC–MS)^18^ identified AURKA, GSK3A and GSK3B as the shared high-affinity kinases (Supplementary Table S3; Figure 2d). As GSK3B did not meet the relative affinity score criteria (Figure 2c), we next undertook functional screening of GSK3A and AURKA. Employing specific chemical inhibitors for the selected kinases (SB415286 for GSK3A; and MLN8237 (alisertib) for AURKA), we performed the soft agar colony formation assay. To ward-off any non-specific effects, we specifically operated within the dose range of previously established IC_50_ values (SB415286: 78 nM; MLN8237: 1.2 nM). GSK3A inhibition failed to suppress the colony-forming potential in both HMEL-B/M and HMEL-B cells (Figure 2e; Supplementary Figures 2B and C). In contrast, nicely reconciling our previous observation from the initial screen with midostaurin, AURKA inhibitor (subsequently referred to as AURKA-i) significantly suppressed the colony-forming potential of both HMEL-B and HMEL-B/M cells (Figure 2e; Supplementary Figures 2B and C). Further supporting the specific inverse relationship between AURKA activity and HMEL-B/M growth in 3D, we observed a clear dose–response association (Supplementary Figure S2D). Corroborating an equal anti-proliferative efficacy of AURKA inhibition in HMEL-B and HMEL-B/M cells, a loss of AURKA function, exhibited a similar anti-proliferative activity. In contrast, GSK3A inhibition elicited a higher anti-proliferative activity in HMEL-B cells (Figure 2f).

We next analyzed the previously generated gene expression data set (GEO accession no. GSE8401)^25^ for *AURKA* transcript levels. *AURKA* expression levels showed highly significant increase with melanoma progression from nevi (*n*=9) to primary (*n*=31) and from primary to metastatic (*n*=52) stage (Figure 2g).

### AURKA is critical for melanoma cell proliferation, survival and migration

**Figure 3:**
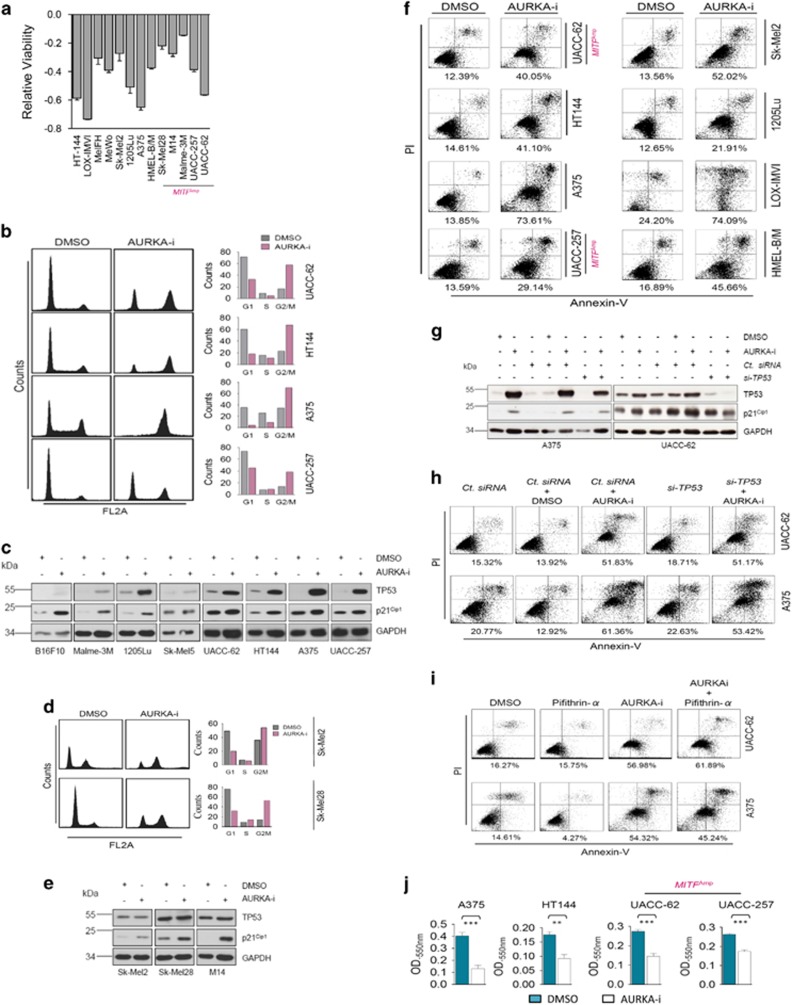
Role of AURKA in melanoma biology. (**a**) Indicated melanoma cell lines were treated with DMSO control or MLN8237 (1 *μ*M) for 48 h followed by measurement of relative viability (*n*=3). (**b**) (Left) Indicated wild-type (wt) *TP53* melanoma cell lines were treated with DMSO control or MLN8237 (1 *μ*M) for 16 h followed by cell cycle analysis. (Right) Quantification of the cell cycle distribution. (**c**) Immunoblotting-based analysis of TP53 and p21^Cip1^ in the indicated wt *TP53* melanoma cell lines upon treatment with DMSO control or MLN8237 (1 *μ*M) for 48 h. (**d**) (Left) Cell cycle analysis of mut-*TP53* Sk-Mel2 and Sk-Mel28 cells upon treatment with DMSO control or MLN8237 (1 *μ*M) for 16 h. (Right) Quantification of the cell cycle distribution. (**e**) Indicated mut-*TP53* melanoma cell lines were treated with DMSO control or MLN8237 (1 *μ*M) for 48 h followed by assessment of TP53 and p21^Cip1^ levels by immunoblotting. (**f**) Indicated melanoma cell lines were treated with DMSO control or MLN8237 (1 *μ*M) for 48 h followed by Annexin V/propidium iodide (PI) staining. Percentages on the bottom correspond to the early apoptotic (Annexin V positive)+late apoptotic (Annexin V+PI positive) cells. (**g**) A375 and UACC-62 cells were treated as indicated for 48 h followed by immunoblotting-based assessment of TP53 and p21^Cip1^ levels. MLN8237 (1 *μ*M) was used. (**h**) A375 and UACC-62 cells were treated as indicated for 48 h followed by Annexin V/PI staining. MLN8237 (1 *μ*M) was used. (**i**) A375 and UACC-62 cells were treated as indicated for 48 h followed by Annexin V/PI staining. Pifithrin-*α* and MLN8237 were used at 10 *μ*M and 1 *μ*M, respectively. (**j**) Soft-agar colony formation assay with the indicated melanoma cell lines treated with DMSO control or MLN8237 (40 nM) for 7 days (*n*=3). Quantification was performed as explained in the methods section. Error bars indicate ±S.D.; **P⩽0.01, ***P⩽0.001

To further understand the role of AURKA in melanoma cell biology, we investigated its requirement in cell viability, utilizing a large panel of melanoma cell lines that encompassed the entire gamut of major melanoma-associated molecular alterations (Supplementary Table S4). AURKA inhibition significantly compromised the viability of all tested cell lines, irrespective of the underlying genetic alterations (Figure 3a).

Consistent with earlier observations,^26^ abrogation of AURKA function triggered a massive accumulation of melanoma cells in G2/M phase (Figure 3b). All tested melanoma lines harbored a functional wild-type (wt)-*TP53* and showed induction of TP53 expression upon AURKA inhibition (Figure 3c). Confirming the transcriptional integrity of induced TP53, we also observed induction in p21^Cip1^ levels (Figure 3c). Interestingly, in contrast to other wt-*TP53* cell lines, Sk-Mel5 cells did not show a clear TP53 or p21^Cip1^ induction and the levels of TP53 protein appeared quite low in B16F10 cells. To conclusively address TP53 requirement in AURKA-i-mediated G2/M arrest, we extended this analysis to two additional mut-*TP53* melanoma cell lines Sk-Mel2 and Sk-Mel28. Interestingly, AURKA inhibition in these cells also triggered a G2/M cell cycle arrest (Figure 3d). Even so, AURKA inhibition failed to induce TP53 levels in the cells harboring mutant *TP53*, quite remarkably, both tested cell lines showed induction in p21^Cip1^ levels (Figure 3e), thus explaining the observed G2/M arrest.

Furthermore, all tested melanoma lines exhibited apoptotic cell death upon AURKA inhibition (Figure 3f; Supplementary Figure S3A). Increased levels of apoptotic protease-activating factor-1 (APAF-1; Supplementary Figure S3B) further substantiated the apoptotic nature of cell death. The apoptotic cell death in mut-*TP53* (MeWo, Sk-Mel2, M14 and Sk-Mel28) and dominant-negative TP53 harboring HMEL-B/M cells excluded the potential requirement of a functional TP53. To conclusively evaluate TP53 requirement in melanoma cells that harbored wt*-TP53*, we adopted a twofold approach, (1) *si-TP53*-mediated rescue of AURKA inhibition-associated TP53 and p21^Cip1^ induction (Figures 3g and h), and (2) TP53 inhibitor Pifithrin-*α*-mediated suppression of TP53 transcriptional activity^27^ (Figure 3i). Although both approaches conferred only a very subtle rescue in AURKA-i-mediated apoptosis in A375 cells, no relief was observed in UACC-62 cells. This hinted towards contextual utilization of additional pro-apoptotic signals that purportedly bypass TP53 requirement.^27^ Because BRAF(V600E)-positive melanomas very frequently develop resistance to targeted BRAF signaling inhibitors, we additionally tested the potential benefit of AURKA-i in melanoma cell lines (451Lu_BR and WM983B_BR) that had developed resistance to BRAF inhibitor PLX-4032. Remarkably, in both the tested resistant cell lines, AURKA inhibition triggered extensive apoptotic cell death (Supplementary Figure S3C). Importantly, human dermal fibroblast FB2003 were quite refractory to MLN8237 treatment (Supplementary Figures 3D and E). Lastly, we tested the growth of melanoma cells, including the *MITF*^Amp^ UACC-62 and UACC-257 in soft agar colony formation assay. Consistent with the data above, AURKA-i compromised the growth of these cells in 3D (Figure 3j). Furthermore, consistent with a report in ovarian cancer cells,^28^ AURKA function was critical to the migration of the tested melanoma cell lines, including the *MITF*^Amp^ UACC-62 and UACC-257 cells (Supplementary Figures S4A and B).

### AURKA inhibition elicits a MAPK-mediated resistance program

**Figure 4:**
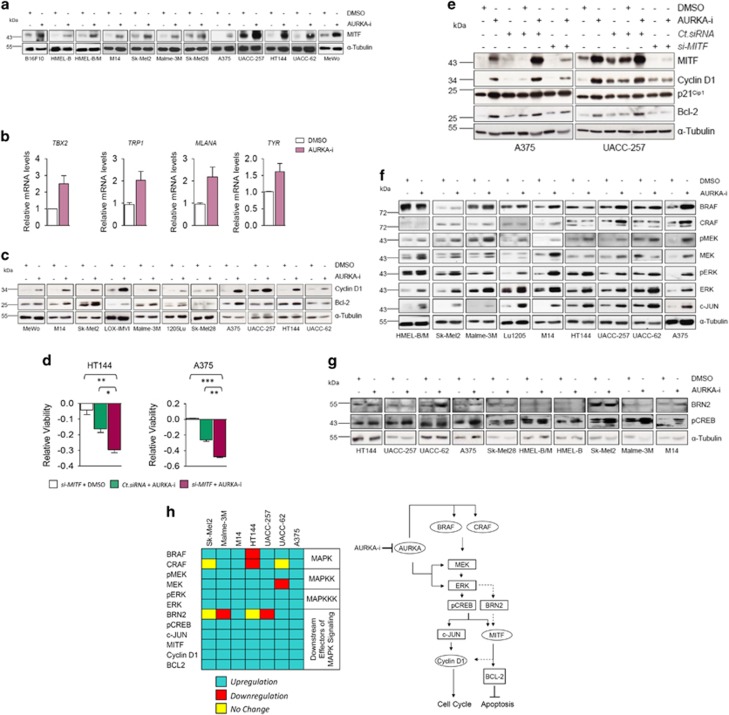
MAPK signaling-elicited resistance program. (**a**) Indicated melanoma cell lines were treated with DMSO control or MLN8237 (1 *μ*M) for 48 h followed by immunoblotting for MITF. (**b**) UACC-62 cells were treated with DMSO control or MLN8237 (1 *μ*M) for 18 h followed by qRT-PCR based transcript analysis for the indicated molecules (*n*=2). The representative experiment is shown. (**c**) Indicated melanoma cell lines were treated for 48 h with DMSO control or MLN8237 (1 *μ*M) followed by the assessment of Cyclin D1 and Bcl-2 levels by immunoblotting. (**d**) HT144 and A375 cells were transfected with *si-MITF*. After 24 h MLN8237 (1 *μ*M) was added for another 24 h. After a total of 48 h relative viability was assessed (*n*=3). (**e**) A375 and UACC-257 cells were treated as indicated followed by immunoblotting for MITF, Cyclin D1, p21^Cip1^ and Bcl-2. MLN8237 used at 1 *μ*M concentration. (**f**) Indicated melanoma cell lines were treated with DMSO control or MLN8237 (1 *μ*M) for 48 h followed by immunoblotting for the indicated molecules. (**g**) Indicated melanoma cell lines were treated with DMSO control or MLN8237 (1 *μ*M) for 48 h followed by immunoblotting for BRN2 and p(Ser-133)CREB1. (**h**) (Left) Box diagram summarizing the expression changes in MAPK signaling and the downstream components in the indicated cell lines upon AURKA inhibition. (Right) Schematic representation of AURKA inhibitor-mediated MAPK signaling-elicited potential resistance program. All error bars indicate ±S.D.; *P⩽0.05, **P⩽0.01, ***P⩽0.001

With its ability to circumvent MITF-associated intrinsic drug resistance, it remained to be seen whether the suppression of AURKA function downregulated MITF expression. Surprisingly, AURKA inhibition induced MITF expression in all tested melanoma cell lines (Figure 4a; Supplementary Figures S5A and B). As there could be a disconnection between MITF expression levels and its transcriptional activity, we also analyzed the expression of MITF target genes *TBX2*, *TRP1* (*TYRP1*), *MLANA* and *TYR*. Providing credence to the transcriptional integrity of upregulated MITF, we observed induction of all the tested MITF transcriptional targets (Figure 4b). Additionally, the induction of other known MITF targets *CDKN1A* (p21^Cip1^) and *CDKN1B (*p27^Kip1^)^29^ (Figures 3c and e; Supplementary Figure S5C) further substantiated the functional nature of induced MITF. These molecular events suggested a MITF-mediated anti-proliferative program.^20^ However, the observed AURKA-i-mediated induction of *CCND1* (Cyclin D1) and Bcl-2, instead, argued for a MITF-mediated potential resistance program^9^ (Figure 4c; Supplementary Figure S5D). To conclusively understand the functional significance of induced MITF and the associated transcriptional program in AURKA-i-mediated anti-proliferative response, we tested melanoma cell viability in response to AURKA-i or *si-MITF,* or a combination thereof. Interestingly, although *MITF* knockdown by itself failed to bear any significant impact on melanoma cell viability, its combination with AURKA-i greatly potentiated the latter's anti-proliferative activity (Figure 4d). These effects coincided with the ability of *si-MITF* to significantly alleviate AURKA inhibition-associated Cyclin D1 and Bcl-2 induction (Figure 4e). However, the failure of *MITF* knockdown to overcome p21^Cip1^ induction (Figure 4e), together with our data showing an intact TP53-p21^Cip1^ axis in wt *TP53* melanoma cells (Figure 3g) excluded a MITF-p21^Cip1^ wiring. Altogether, these data suggest AURKA-i-associated MITF induction as a potential resistance program.

Although MAPK signaling has been shown to promote MITF transcriptional activity through its phosphorylation, this post-translational modification also promotes MITF's proteasome-mediated degradation.^21^ Additionally, MAPK signaling counterbalances this kinase function-effected MITF degradation through the latter's BRN2-mediated transcription.^21^ To test whether a MAPK signaling-effected transcription program could potentially explain MITF induction in response to AURKA inhibition, we first analyzed the expression and/or activity changes of key MAPK signaling components (ERK, MEK, BRAF, CRAF and c-JUN) in a panel of human melanoma cell lines. AURKA inhibition induced both the expression and activity of ERK (Figure 4f). Furthermore, while an increased phosphorylation of MEK was observed in all the tested cell lines, except UACC-62, all cell lines also exhibited elevated MEK expression levels. Further probing into the expression changes of the upstream MAPKs (BRAF and CRAF) showed increased BRAF (seven of the nine cell lines) and CRAF levels (four of the nine cell lines). Documenting the downstream consequential nature of this induced MAPK signaling, in addition to MITF, Cyclin D1 and Bcl-2 induction (Figures 4a and c), we also observed c-JUN upregulation (Figure 4f). Altogether, these molecular changes suggested a MAPK signaling-mediated program of MITF upregulation and associated resistance. Therefore, we next interrogated a potential BRN2-mediated induction of MITF, downstream of the activated MAPK signaling cascade. Interestingly, in spite of a clear MAPK signaling hyperactivation in all tested cell lines (Figure 4f), only a few exhibited a corresponding induction in BRN2 levels (Figure 4g).

In response to changes in the cellular cyclic adenosine mono-phosphate (cAMP) levels, followed by protein kinase A (PKA)-mediated phosphorylation, cAMP-response element-binding protein (CREB) has also been shown to induce MITF expression.^9, 13^ CREB phosphorylation at Ser133 by ERK^30^ has also been reported to transcriptionally activate it, thus leading to the upregulation of c-JUN, Cyclin D1 and proliferation program thereupon.^31^ Moreover, p(Ser133)-CREB transcriptionally regulates MITF.^32^ Therefore, our results, in the light of above reports point to CREB as a mediator of MITF upregulation downstream of AURKA inhibition-mediated MAPK signaling induction. Consistently, in contrast to the observations with BRN2, AURKA inhibition induced p(Ser133)-CREB levels in all the tested melanoma cell lines (Figure 4g).

Taken together, these data pre-emptively reveal a potentially significant mechanism of MAPK signaling-mediated early acquired resistance to AURKA inhibition, which involves an upregulation of pERK/ERK and the associated downstream pro-proliferative (c-JUN, Cyclin D1) and anti-apoptotic (MITF, Bcl-2) molecular signature (Figure 4h).

### Co-Targeting AURKA and MAPK signaling-elicited resistance program

**Figure 5:**
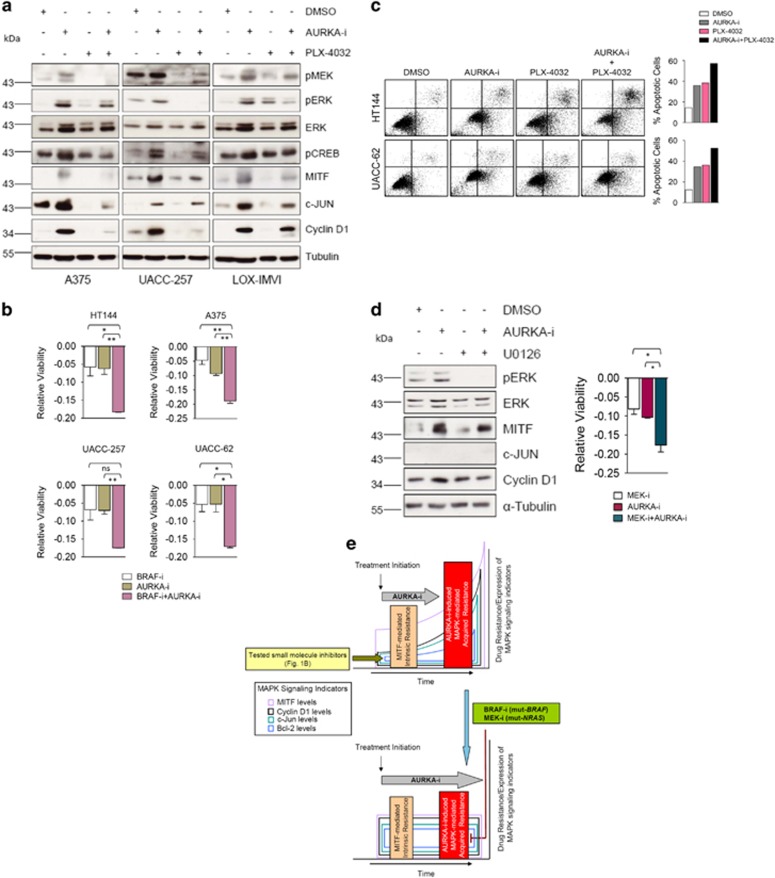
Combinatorial AURKA and MAPK targeting in melanomas. (**a**) A375, UACC-62 and LOX-IMVI cells were treated as indicated for 48 h followed by immunoblotting-based analysis of the indicated proteins. MLN8237 (1 *μ*M) and PLX-4032 (1 *μ*M) were used. (**b**) HT144, A375, UACC-257 and UACC-62 cells were treated as indicated for 48 h followed by the assessment of relative cell viability (*n*=3). MLN8237 (10 nM) and PLX-4053 (10 nM) were used. (**c**) (Left) HT144 and UACC-62 cells were treated as indicated for 48 h followed by Annexin V/PI staining. MLN8237 (0.5 *μ*M) and PLX-4053 (1 *μ*M) were utilized. (Right) Bar diagrams showing the corresponding quantification. The representative experiment is shown. (**d**) (Left) NRAS(Q61R)-mutated Sk-Mel2 cells were treated as indicated for 48 h followed by western blotting for the indicated proteins. MLN8237 (1 *μ*M) and U0126 (1 *μ*M) were used. (Right) Sk-Mel2 cells were treated as indicated for 48 h followed by the measurement of relative viability (*n*=3). MLN8237 (100 nM) and U0126 (100 nM) were used. (**e**) (Upper panel) Unlike all the tested small molecule inhibitors (Figure 1b), AURKA inhibition overcomes MITF-mediated intrinsic drug resistance. However, after some time lapse, AURKA inhibition triggers MAPK signaling activation through enhanced expression/activity of several MAPK signaling components (Figure 4). MAPK signaling induction in-turn upregulates downstream proliferative/drug resistance molecular signature, including MITF, c-Jun, Cyclin D1 and Bcl-2 induction. (Lower panel) A concomitant treatment with MAPK inhibitors (BRAF inhibitor for mut-*BRAF* and MEK inhibitor for mut-*NRAS*) successfully relieves the induction of MAPK signaling components and the associated downstream expression signature; overrides the acquired resistance build-up; and potentiates the anti-proliferative efficacy of AURKA inhibition. All error bars indicate ±S.D.; ns, non-significant; *P⩽0.05, **P⩽0.001

The robust build-up of MAPK signaling activity and the associated downstream proliferative/pro-survival molecular manifestations (Figure 4) alluded to a potential mechanism of resistance to AURKA inhibition. This argued for a combinatorial targeting of AURKA and MAPK signaling as a viable therapeutic option. Imparting further support for this therapeutic regimen, a concomitant inhibition of AURKA and BRAF(V600E), utilizing PLX-4032, nicely alleviated the MAPK signaling induction and the associated resistance signature, including upregulated Cyclin D1 and c-JUN levels (Figure 5a). Nicely translating into functional significance, a combined inhibition of BRAF and AURKA achieved heightened anti-proliferative activity (Figure 5b), and also exhibited more efficient apoptotic cell killing (Figure 5c). Similarly, MEK inhibition in mut-NRAS(Q61R) harboring Sk-Mel2 cells alleviated AURKA inhibition-mediated induction of MAPK signaling and the associated downstream events (Figure 5d). Consistently, this molecular rescue heightened the anti-proliferative activity of AURKA-i in these cells.

Cumulatively, based on these findings, we propose a model (Figure 5e) detailing the rationale for the dual targeting of AURKA and MAPK signaling in melanomas harboring the constitutively active BRAF(V600E) or NRAS (Q61R).

## Discussion

The target identification approach ‘DEFINIT', developed and utilized in the current study appreciated the destructive liaison between the oncogenic driver BRAF(V600E) and often concomitantly amplified *MITF*^14^ to identify combinatorial MAPK signaling and AURKA inhibition as more effective therapeutic approach in melanoma. This multi-step target identification is inspired by similar yet distinct target identification approaches.^33, 34^

Identification of AURKA as a highly overexpressed gene in late-stage melanoma that overcomes the surveillance function of TP53 by orchestrating its proteasome-mediateddegradation^35^ bears significant implications towards the understanding of molecular mechanisms failing TP53 tumor suppressor activity in this malignancy. Although a recent report suggested the inability of AURKA inhibition to fully switch-on TP53-associated transcriptional function and induce apoptosis,^36^ our studies with multiple wt-TP53 melanoma cell lines suggest AURKA-i fully capable of inducing TP53, its transcription activity and apoptotic cell death. These data are in conformity with previous reports showing induction of TP53-associated transcriptional activity and apoptotic cell death upon AURKA inhibition.^26, 35, 37^ Interestingly, one of the wt-TP53 cell lines (Sk-Mel5), also studied by Vilgelm *et al.*^36^ showed only subtle increase in TP53 and its target p21^Cip1^ levels in our hands. However, remaining seven cell lines investigated in the current study showed clear induction in both TP53 and p21^Cip1^ levels. This underscores the necessity of utilizing multiple cancer cell lines to gain general mechanistic understanding. Furthermore, our data showing AURKA-i-mediated p21^Cip1^ induction and an anti-proliferative activity even in the mut-TP53 cells, while bearing important mechanistic and therapeutic significance could provide basis for observed TP53 dispensability in AURKA-i-mediated apoptosis. Although p73-mediated p21^Cip1^ regulation has previously been proposed in mut-TP53 cells,^38^ additional studies would be required to gain a detailed understanding.

Although our analysis revealed a highly significant disease stage-associated increase in *AURKA* transcript levels, querying the provisional TCGA data (http://www.cbioportal.org/) consisting of 477 cases of cutaneous melanoma returned only six samples with an amplification of the corresponding (20q) locus. Furthermore, analysis of our own array comparative genome hybridization (aCGH) data (GEO accession no. GSE7606)^39^ showed merely ~1.6% and ~0.8% of primary and metastatic samples, respectively, with an accompanying copy number gain. Interestingly, a recent report suggested MAPK signaling-mediated transcriptional induction of Aurora kinase family member Aurora B.^40^ Although this finding together with a highly prevalent melanoma-associated MAPK signaling hyperactivity would argue for a similar mechanism of AURKA regulation, the early procurement of BRAF(V600E) in the evolution of melanoma cells, together with a correspondingly low *AURKA* levels in melanocytic nevi (Figure 2g) would argue against such regulatory axis. In contrast, a cell cycle-dependent regulation, as previously reported for a functionally related Polo-Like Kinase 1^41^ might be a more conceivable mechanism of elevated AURKA expression levels with melanoma progression. Furthermore, NEDD9, which is frequently overexpressed in metastatic melanoma, has been shown to interact with, activate and stabilize AURKA.^42, 43^ All in all, further studies are needed to gain a better understanding of AURKA expression deregulation in late-stage melanomas.

Our data documenting the ability of AURKA inhibition to counter/bypass a significant intrinsic resistance associated with MITF to a range of therapeutics, while failing to downregulate MITF expression, argues for AURKA's point of operation either independent or downstream of the growth mechanisms regulated by MITF. With MITF's credentials as a transcription factor that regulates G1–S phase transition through a transcriptional control of *CDK2*, *CDKN2A*, *CDKN1A* and *CDKN1B,*^44^ AURKA's known point of operation at mitotic phase provides credence to the latter possibility. Furthermore, our previous demonstration of PLK-1 as a potential therapeutic target in melanoma,^41^ together with the ability of PLK-1 inhibition to override MITF-mediated intrinsic resistance (Supplementary Figure S6) suggest mitotic kinases as potential avenues for therapeutic intervention in melanoma.

Detailed reports witnessing the reactivation of PKA-CREB-MITF axis in melanoma patients relapsing from vemurafenib,^9^ and the revelation of a MITF-dependent shift in metabolic preference from anaerobic glycolysis to oxidative phosphorylation in melanoma cells developing vemurafenib resistance^17^ underscore MITF's acquired drug resistance credentials. To the best of our knowledge, the presented data, for the first time, unveils the recruitment of MAPK signaling, including MITF induction, as a mechanism of immediate acquired resistance to AURKA inhibition. MEK-ERK signaling activation upon the introduction of BRAF(V600E), by orchestrating the phosphorylation of MITF, promotes its proteasomal degradation.^20, 21^ On the other hand, MAPK signaling, through the regulation of BRN2 and CREB activity is also required for maintaining MITF levels.^21, 32^ Our results showing AURKA-i-mediated activation of MAPK signaling, induction of p-CREB levels and the consequential upregulation of MITF expression, suggests the latter signaling mechanism as the predominant player in the setting of AURKA inhibition. AURKA inhibitor MLN8237 has already entered phase III clinical trials for different cancers and phase-II trials in melanoma patients. MAPK cascade induction in human melanoma cells could therefore jeopardize the success of AURKA interference approaches. Therefore, our studies, pre-emptively demonstrating the ability of a concomitant MAPK pathway inhibition to relieve AURKA-i-initiated MAPK signaling-mediated resistance program, recommend a highly promising combination regimen. Although a recent study has also noted the beneficial effects of AURKA and BRAF inhibitor combination,^45^ the mechanistic rationale for this combination, as elucidated in our studies, had been lacking in this previous work.

Notably, whereas BRAF inhibitors have been shown to induce only a change in the activity of the MAPK signaling components, AURKA inhibition triggers both expression and activity changes in MAPK signaling proteins.

To conclude, this study, whilst potentially bearing fundamental implications for the application of AURKA inhibitors as melanoma therapeutics, describes a common framework for future target discovery, whereby the functional significance of cooperating oncogenic events is addressed at the outset.

## Materials and Methods

### Cell culture and reagents

All melanoma cell lines (Supplementary Table S4) were cultured in RPMI (Invitrogen, Vienna, Austria), supplemented with 10% FCS (Invitrogen). Primary human melanocytes transduced with hTERT, p53DD, CDK4(R24C) (primary melanocytes/hTERT/CDK4(R24C)/p53DD) resulting in immortalized melanocytes (HMEL cells), with ectopically expressed BRAFV600E (HMEL-B) or with ectopically expressed BRAFV600E and HA-MITF (HMEL-B/M) have been previously described.^14^ All utilized small molecule inhibitors are summarized in Supplementary Table S1. MLN8237 was bought from Selleckchem (Houston, TX, USA). Midostaurin and Sunitinib were obtained from LC Laboratories (Woburn, MA, USA).

### Antibodies

Phospho-(Ser218/Ser222)-MEK1/2, MEK1 (C-18), p53 (TP53) (DO-1), p21^Cip1^ (H-164), Bcl-2 (100), Raf-1 (C-12), Raf-B (C-19), APAF-1, Phospho-(Ser133)-CREB-1, BRN2 (C20) and GAPDH (FL-335) from Santa Cruz Biotechnology (Santa Cruz, CA, USA). c-Jun (60A8), Phospho-ERK1/2 (Thr202/Tyr204), ERK1/2 from Cell Signaling Technology (Danvers, MA, USA). MITF(C5) from Abcam (Cambridge, UK). Cyclin D1 from BD Biosciences (Schwechat, Austria). *α*-Tubulin antibody from Calbiochem (Darmstadt, Germany).

### DEFINIT

Previously described immortalized human melanocytes (HMEL-B and HMEL-B/M) with isogenic background^14^ were targeted with multi-kinase inhibitors midostaurin and sunitinib, and tested for anchorage-independent growth. In view of the common spectrum of the kinase targets of the inhibitors, the differential effect of the drugs against MITF-expressing HMEL-B/M cells was exploited for further analysis, utilizing drug pull-down-based proteomics.^18^ Using recently published 1D-LC–MS and iTRAQ labeling combined with gel-free 2D-LC-MS data,^18^ the kinases with differential affinity for midostaurin were identified. This was followed by functional screening of the identified kinases in soft agar colony formation assay and finally gene expression-disease stage correlation, utilizing previously published gene expression data from melanocytic nevi, primary and metastatic melanoma samples (GEO accession no. GSE8401).^25^

### Gene expression profiling

Tissue sampling and gene expression profiling were previously performed using Affymetrix U133A microarray platform as described earlier.^25^ The data have been deposited in the National Center for Biotechnology Information GEO^46^ and are accessible through GEO Series accession no. GSE8401.

### Viability (MTT) assay

MTT assay was performed as described previously.^41^ Relative viability was calculated as viability change relative to solvent-treated cells; viability of solvent-treated cells was set to zero. Resistivity index was calculated using the formula ((relative viability of HMEL-B)/(relative viability of HMEL-B/M)); greater the value of resistivity index, higher the resistance of HMEL-B/M cells to the specified treatment in comparison to HMEL-B cells.

### RNA interference

siRNA transfections using *TP53*-siRNA (sc-416469-NIC-2), *MITF*-siRNA (110566) and negative control siRNA (AM4636) (Life Technologies, Carlsbad, CA, USA) were performed employing Lipofectamine 2000 transfection reagent (Invitrogen) as per manufacturer's instructions.

### Cell cycle analysis

Cell-Cycle analysis was performed as previously described.^41^ Hypodiploid (necrotic/apoptotic) (Sub-G1 phase), diploid (G1/G0 phase), hyperdiploid (S phase) and tetraploid (G2/M) cell populations were quantified using CellQuest software (BD Biosciences).

### Annexin V/PI staining

Annexin V/PI-based apoptosis detection and quantification was performed as previously described.^41^

### Anchorage-independent growth

Short-term anchorage-independent growth assay was assessed in triplicates in a fluorescence-based 1 week assay as previously described.^47^ The cultures were performed either with 40 nM MLN8237 or DMSO control. After 7-day incubation, colonies were stained with AlamarBlue (Invitrogen) according to the manufacturer's instructions. Calorimetric readout was performed using a multi-well plate reader at 570 and 600 nm reference wavelength.

Long-term anchorage-independent growth assay was carried out in 24-well plate format as previously described with some modifications.^33^ Briefly, 4x10^4^ cells were seeded as above for 4 weeks. The medium was weekly replenished with the drugs being investigated. Images were acquired with Alpha Imager (Biozym, Vienna, Austria).

### Transwell migration assays

Transwell migration assays were performed as previously described.^33^ The cultures contained either 30 nM MLN8237 or DMSO as control. Following incubation for 20 h, cells on the bottom side of the insert membranes were fixed and stained using Kwik-Diff Staining kit (Thermo Fisher Scientific, Waltham, MA, USA) according to the manufacturer's instructions. Migrated cells were quantified by counting of six randomly selected microscopic fields.

### Immunoblotting

Western Blotting was performed as previously described.^41^ *α*-Tubulin or GAPDH staining was used as a control for equal sample loading.

### RNA extraction and quantitative real-time PCR

Total cellular RNA was extracted using TRI Reagent (Sigma-Aldrich, St. Louis, MO, USA) according to the manufacturer's protocol, and 1 *μ*g per sample was subjected to reverse transcription, using Superscript II Reverse Transcriptase (Invitrogen). TaqMan gene expression assays for *TBX2, TRP1, MLANA, TYR, CDKN1A, CDKN1B, CCND1, MITF* and *ACTIN-B1* (Life Technologies; *TBX2*: Hs00172983, *TRP1*: Hs00167051-m1, *MLANA*: Hs00194133-m1, *TYR*: Hs00165976-m1, *MITF*: Hs00165156_m1, *CDKN1A*: Hs00355782-m1, *CDKN1B*: Hs00153277-m1 *CCND1*: Hs00277039-m1, *ACTIN-B1*: Hs99999903-m1). A StepOne Plus qRT-PCR System was used for amplification (2 min 50 °C; 10 min 95 °C; 40 cycles: 15 s 95 °C, 1 min 60 °C) and detection. Reverse transcription-negative controls were always included. For relative quantification of gene expression, the 2−ΔΔCT method was used.

### Statistical analysis

Graphpad prism software 5.0 (Graphpad, La Jolla, CA, USA; http://www.graphpad.com) was used to perform statistical analysis by performing unpaired *t*-test.

## Abbreviations

MITF: microphthalmia-associated transcription factor
AURKA: aurora kinase A
MAPK: mitogen-activated protein kinase
