# Supplementary material for: Overcoming MITF-conferred drug resistance through dual AURKA/MAPK targeting in human melanoma cells
Source: Cell Death Dis. 2016 Mar 10;7(3):e2135–. doi: 10.1038/cddis.2015.369 (PMC4823922; doi:10.1038/cddis.2015.369)
Supplement: Supplementary Information [file cddis2015369x1.ppt]

## Slide 1
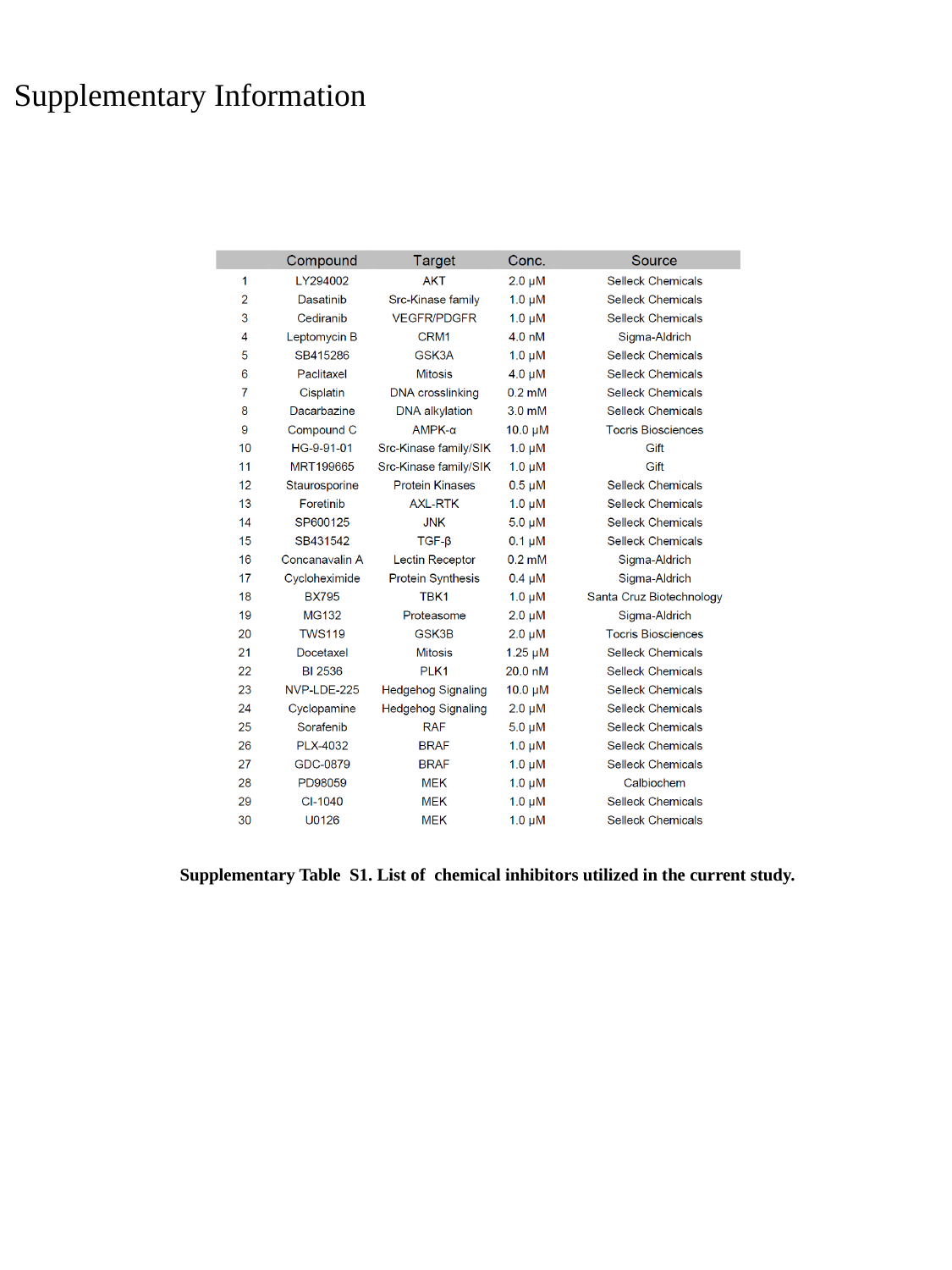

Supplementary Information
Supplementary Table S1. List of chemical inhibitors utilized in the current study.

## Slide 2
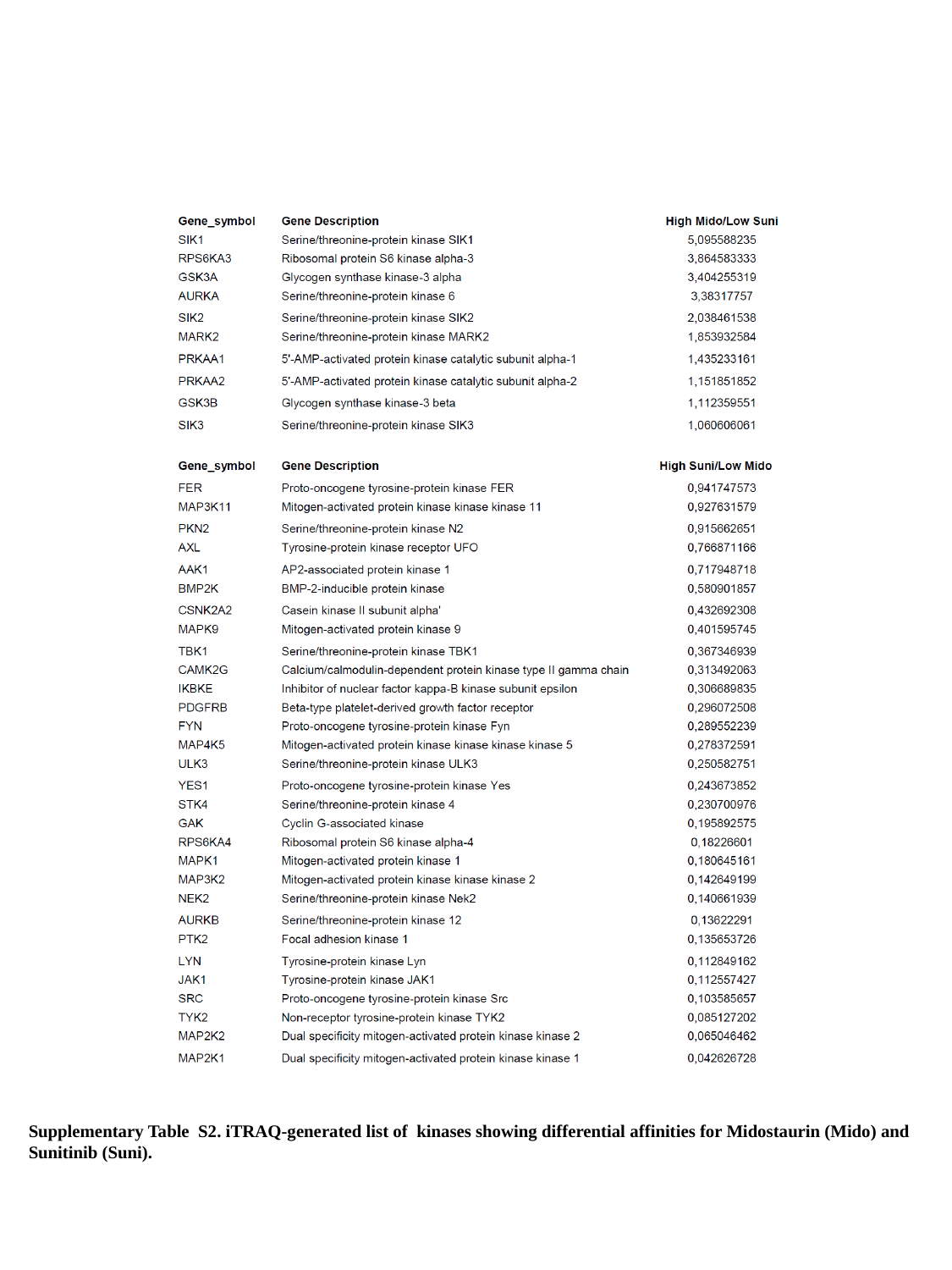

Supplementary Table S2. iTRAQ-generated list of kinases showing differential affinities for Midostaurin (Mido) and Sunitinib (Suni).

## Slide 3
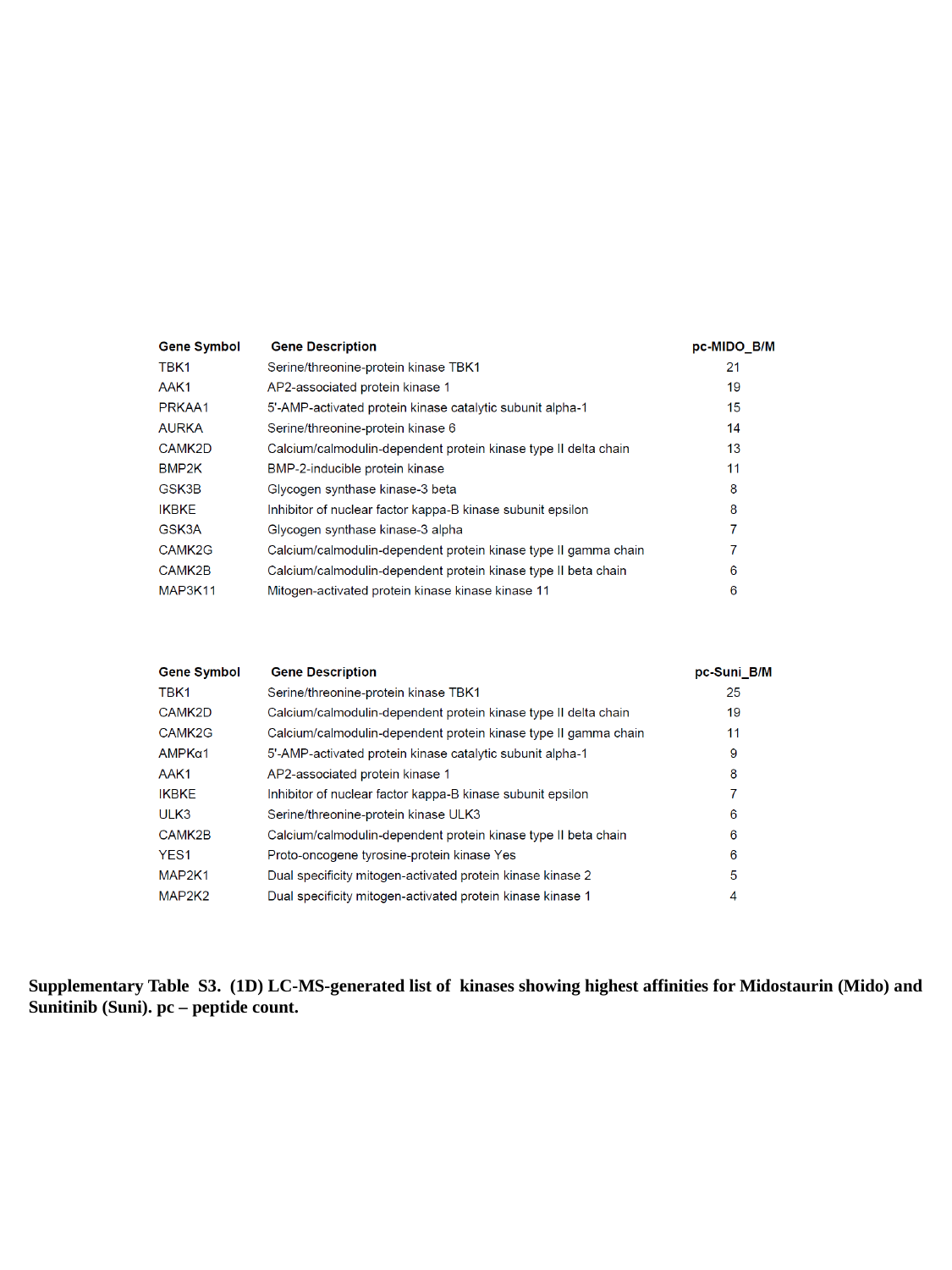

Supplementary Table S3. (1D) LC-MS-generated list of kinases showing highest affinities for Midostaurin (Mido) and Sunitinib (Suni). pc – peptide count.

## Slide 4
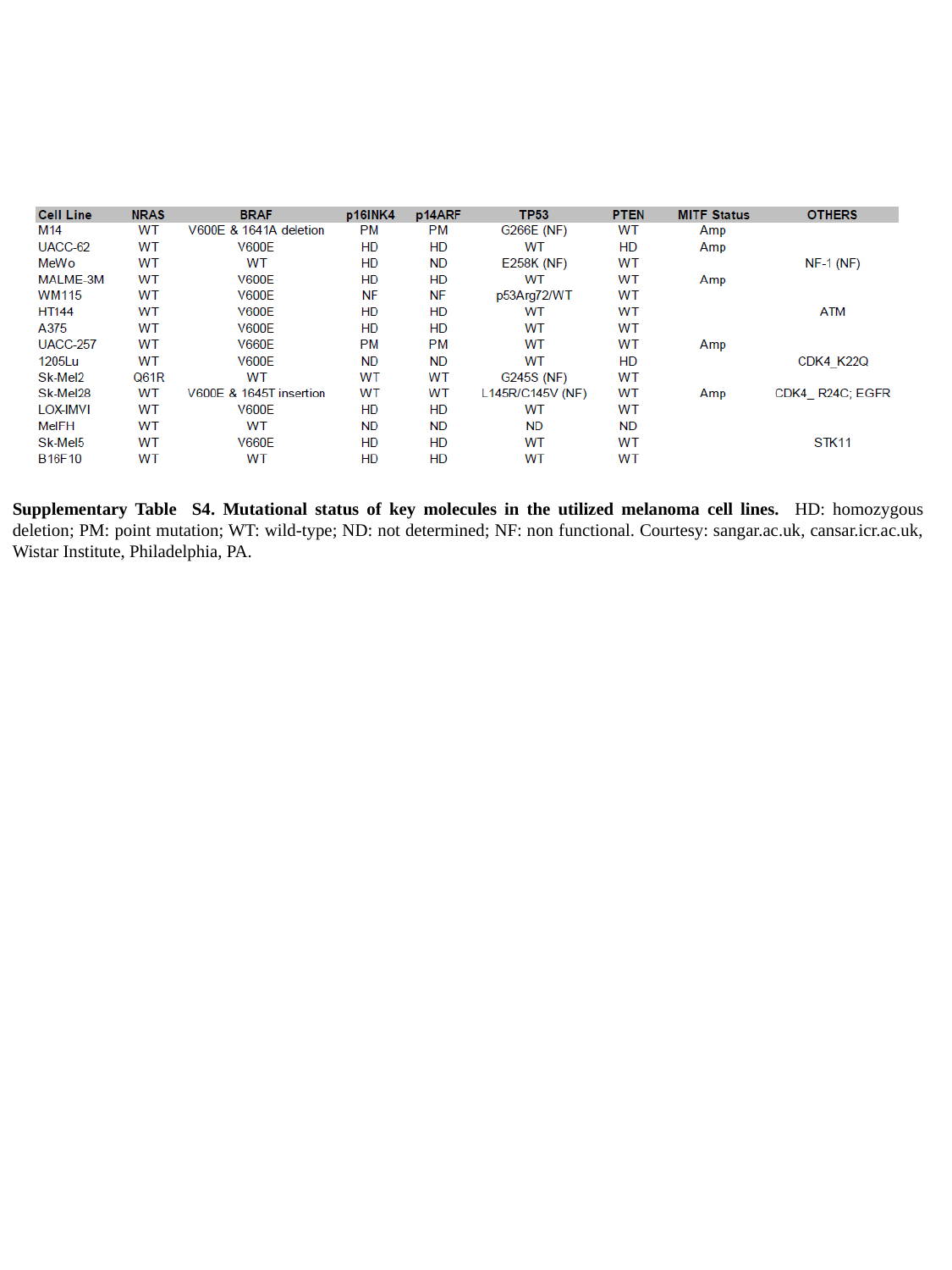

Supplementary Table S4. Mutational status of key molecules in the utilized melanoma cell lines. HD: homozygous deletion; PM: point mutation; WT: wild-type; ND: not determined; NF: non functional. Courtesy: sangar.ac.uk, cansar.icr.ac.uk, Wistar Institute, Philadelphia, PA.

## Slide 5
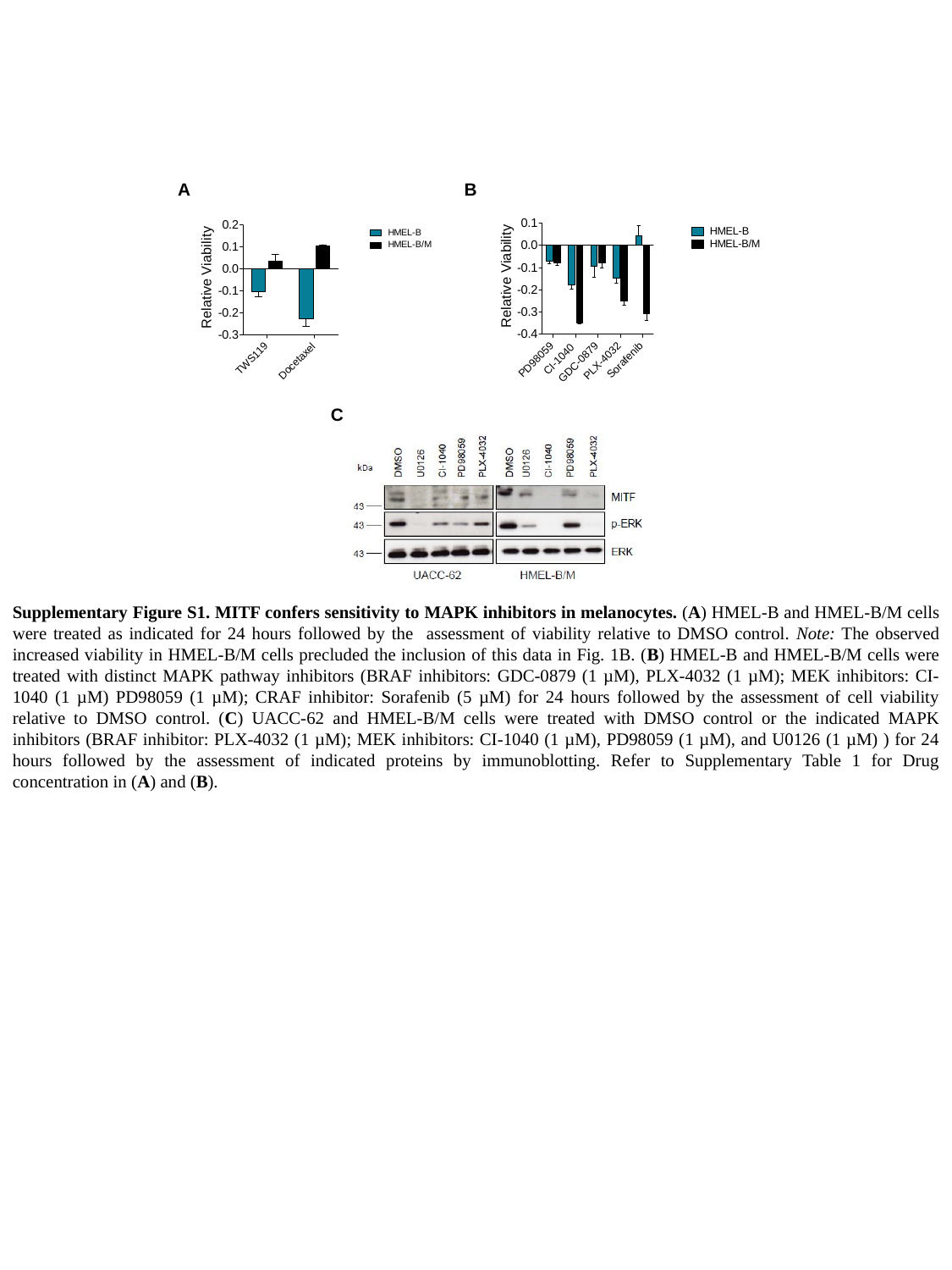

A
B
C
Supplementary Figure S1. MITF confers sensitivity to MAPK inhibitors in melanocytes. (A) HMEL-B and HMEL-B/M cells were treated as indicated for 24 hours followed by the assessment of viability relative to DMSO control. Note: The observed increased viability in HMEL-B/M cells precluded the inclusion of this data in Fig. 1B. (B) HMEL-B and HMEL-B/M cells were treated with distinct MAPK pathway inhibitors (BRAF inhibitors: GDC-0879 (1 µM), PLX-4032 (1 µM); MEK inhibitors: CI-1040 (1 µM) PD98059 (1 µM); CRAF inhibitor: Sorafenib (5 µM) for 24 hours followed by the assessment of cell viability relative to DMSO control. (C) UACC-62 and HMEL-B/M cells were treated with DMSO control or the indicated MAPK inhibitors (BRAF inhibitor: PLX-4032 (1 µM); MEK inhibitors: CI-1040 (1 µM), PD98059 (1 µM), and U0126 (1 µM) ) for 24 hours followed by the assessment of indicated proteins by immunoblotting. Refer to Supplementary Table 1 for Drug concentration in (A) and (B).

## Slide 6
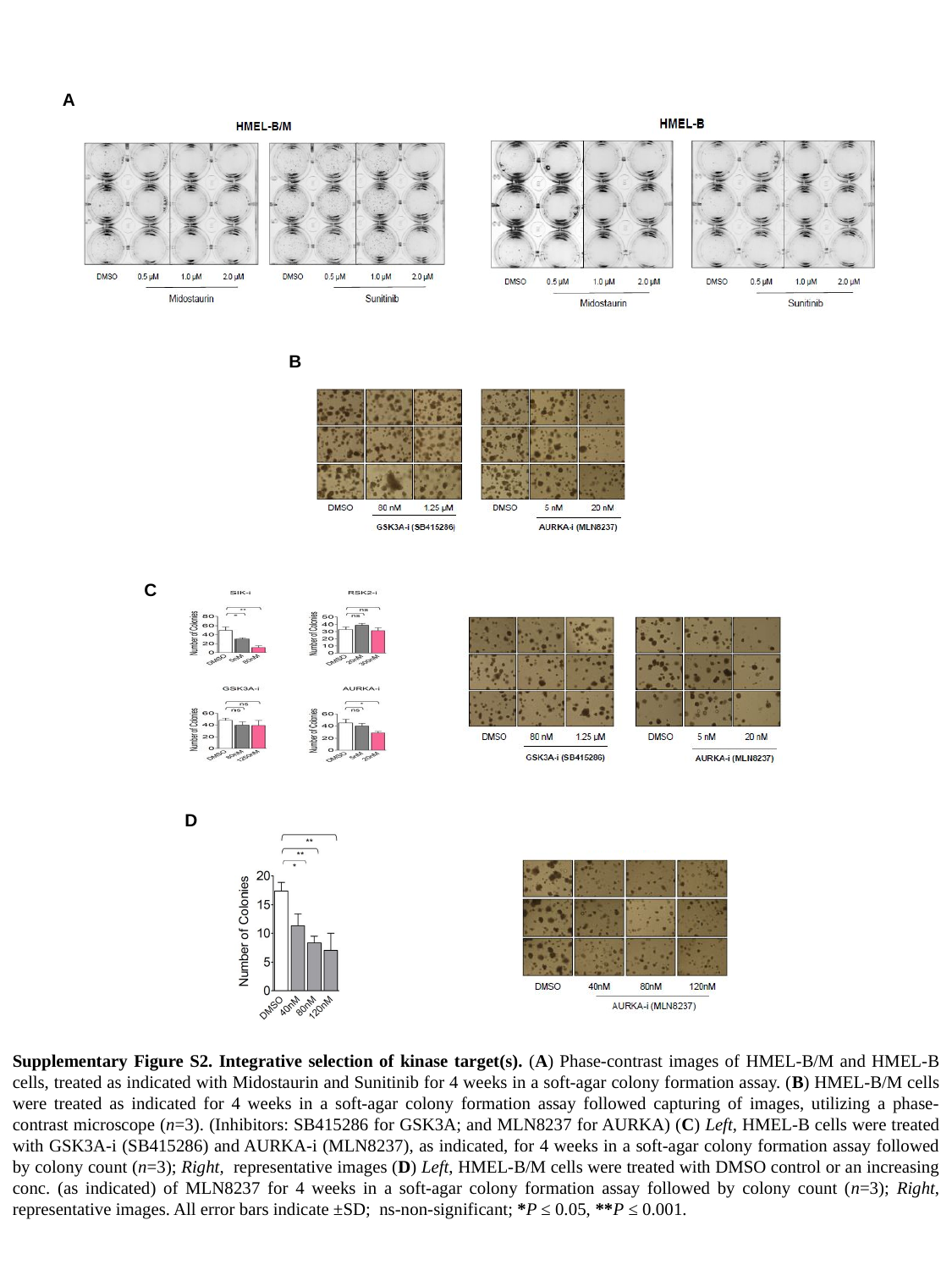

A
B
C
D
Supplementary Figure S2. Integrative selection of kinase target(s). (A) Phase-contrast images of HMEL-B/M and HMEL-B cells, treated as indicated with Midostaurin and Sunitinib for 4 weeks in a soft-agar colony formation assay. (B) HMEL-B/M cells were treated as indicated for 4 weeks in a soft-agar colony formation assay followed capturing of images, utilizing a phase-contrast microscope (n=3). (Inhibitors: SB415286 for GSK3A; and MLN8237 for AURKA) (C) Left, HMEL-B cells were treated with GSK3A-i (SB415286) and AURKA-i (MLN8237), as indicated, for 4 weeks in a soft-agar colony formation assay followed by colony count (n=3); Right, representative images (D) Left, HMEL-B/M cells were treated with DMSO control or an increasing conc. (as indicated) of MLN8237 for 4 weeks in a soft-agar colony formation assay followed by colony count (n=3); Right, representative images. All error bars indicate ±SD; ns-non-significant; *P ≤ 0.05, **P ≤ 0.001.

## Slide 7
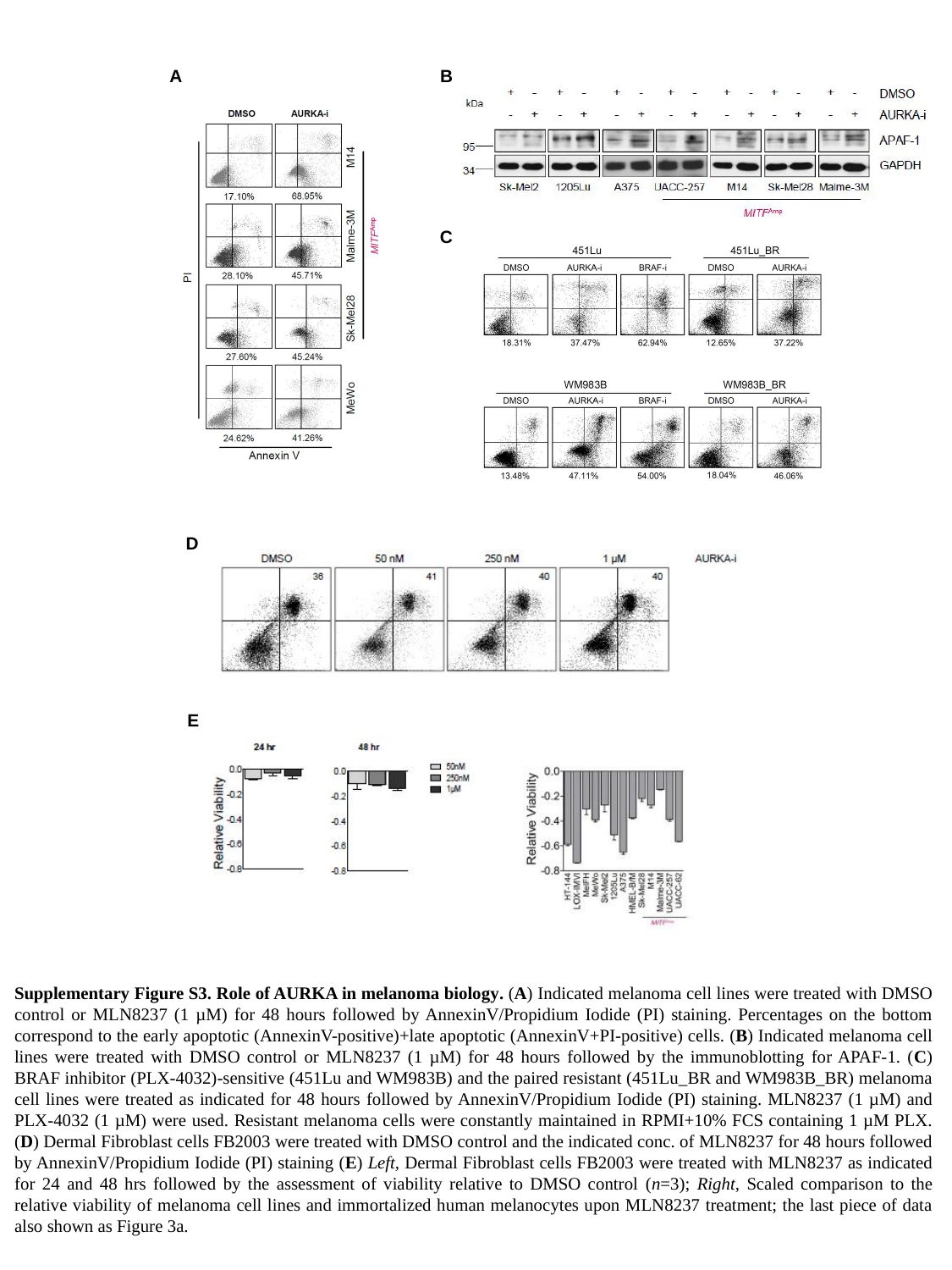

A
B
C
D
E
Supplementary Figure S3. Role of AURKA in melanoma biology. (A) Indicated melanoma cell lines were treated with DMSO control or MLN8237 (1 µM) for 48 hours followed by AnnexinV/Propidium Iodide (PI) staining. Percentages on the bottom correspond to the early apoptotic (AnnexinV-positive)+late apoptotic (AnnexinV+PI-positive) cells. (B) Indicated melanoma cell lines were treated with DMSO control or MLN8237 (1 µM) for 48 hours followed by the immunoblotting for APAF-1. (C) BRAF inhibitor (PLX-4032)-sensitive (451Lu and WM983B) and the paired resistant (451Lu_BR and WM983B_BR) melanoma cell lines were treated as indicated for 48 hours followed by AnnexinV/Propidium Iodide (PI) staining. MLN8237 (1 µM) and PLX-4032 (1 µM) were used. Resistant melanoma cells were constantly maintained in RPMI+10% FCS containing 1 µM PLX. (D) Dermal Fibroblast cells FB2003 were treated with DMSO control and the indicated conc. of MLN8237 for 48 hours followed by AnnexinV/Propidium Iodide (PI) staining (E) Left, Dermal Fibroblast cells FB2003 were treated with MLN8237 as indicated for 24 and 48 hrs followed by the assessment of viability relative to DMSO control (n=3); Right, Scaled comparison to the relative viability of melanoma cell lines and immortalized human melanocytes upon MLN8237 treatment; the last piece of data also shown as Figure 3a.

## Slide 8
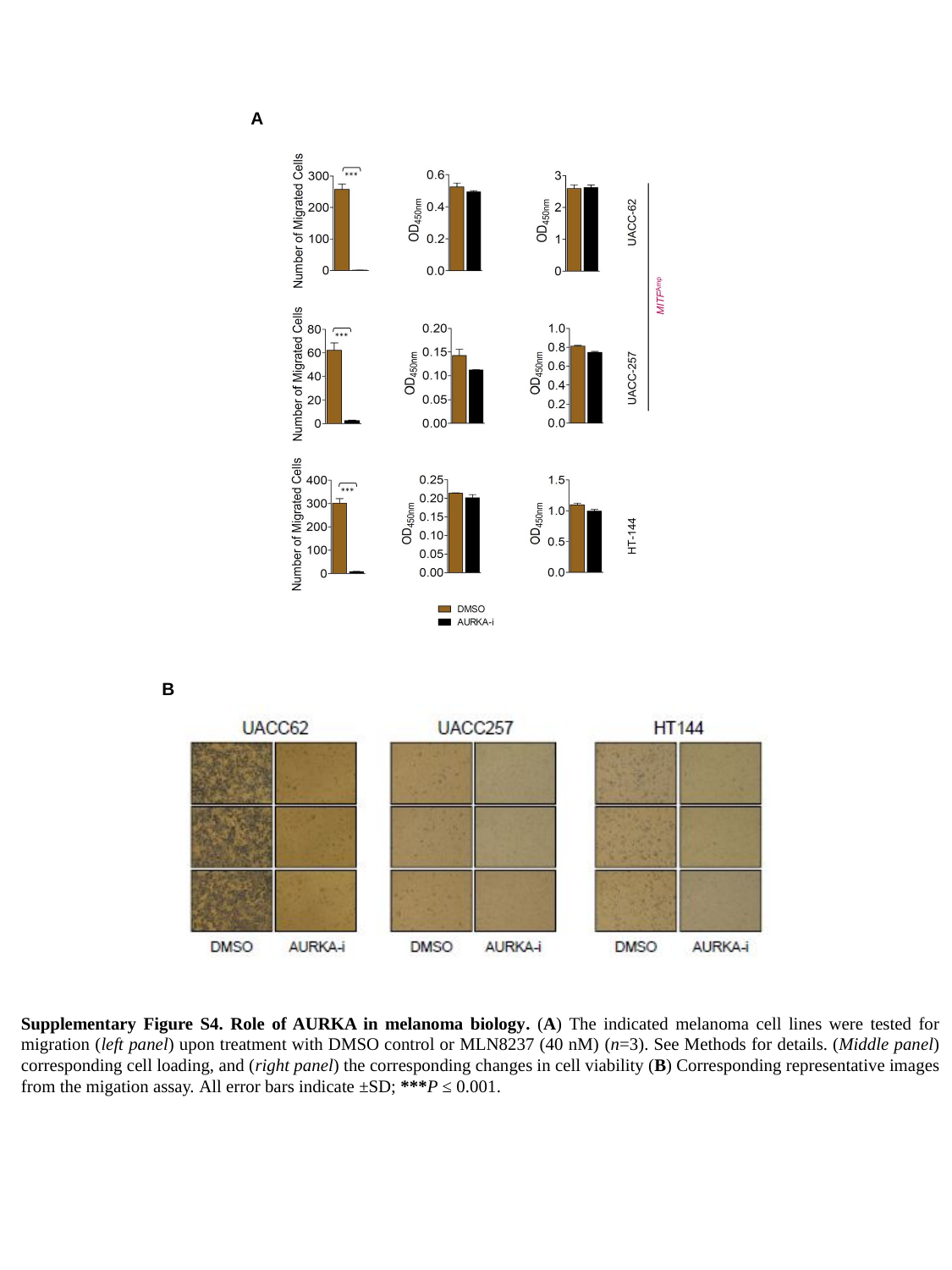

A
B
Supplementary Figure S4. Role of AURKA in melanoma biology. (A) The indicated melanoma cell lines were tested for migration (left panel) upon treatment with DMSO control or MLN8237 (40 nM) (n=3). See Methods for details. (Middle panel) corresponding cell loading, and (right panel) the corresponding changes in cell viability (B) Corresponding representative images from the migation assay. All error bars indicate ±SD; ***P ≤ 0.001.

## Slide 9
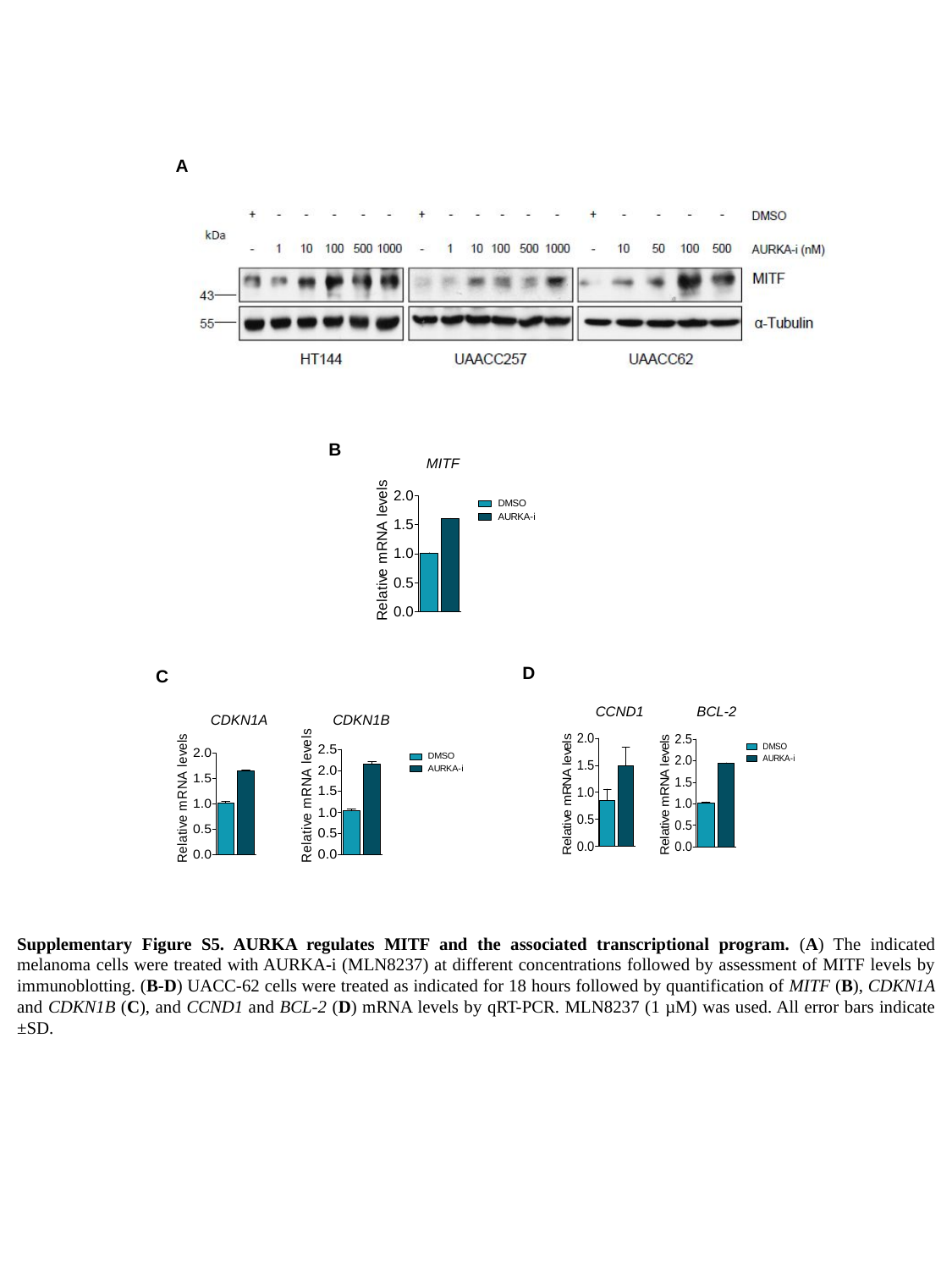

A
B
MITF
D
C
CCND1
BCL-2
CDKN1A
CDKN1B
Supplementary Figure S5. AURKA regulates MITF and the associated transcriptional program. (A) The indicated melanoma cells were treated with AURKA-i (MLN8237) at different concentrations followed by assessment of MITF levels by immunoblotting. (B-D) UACC-62 cells were treated as indicated for 18 hours followed by quantification of MITF (B), CDKN1A and CDKN1B (C), and CCND1 and BCL-2 (D) mRNA levels by qRT-PCR. MLN8237 (1 µM) was used. All error bars indicate ±SD.

## Slide 10
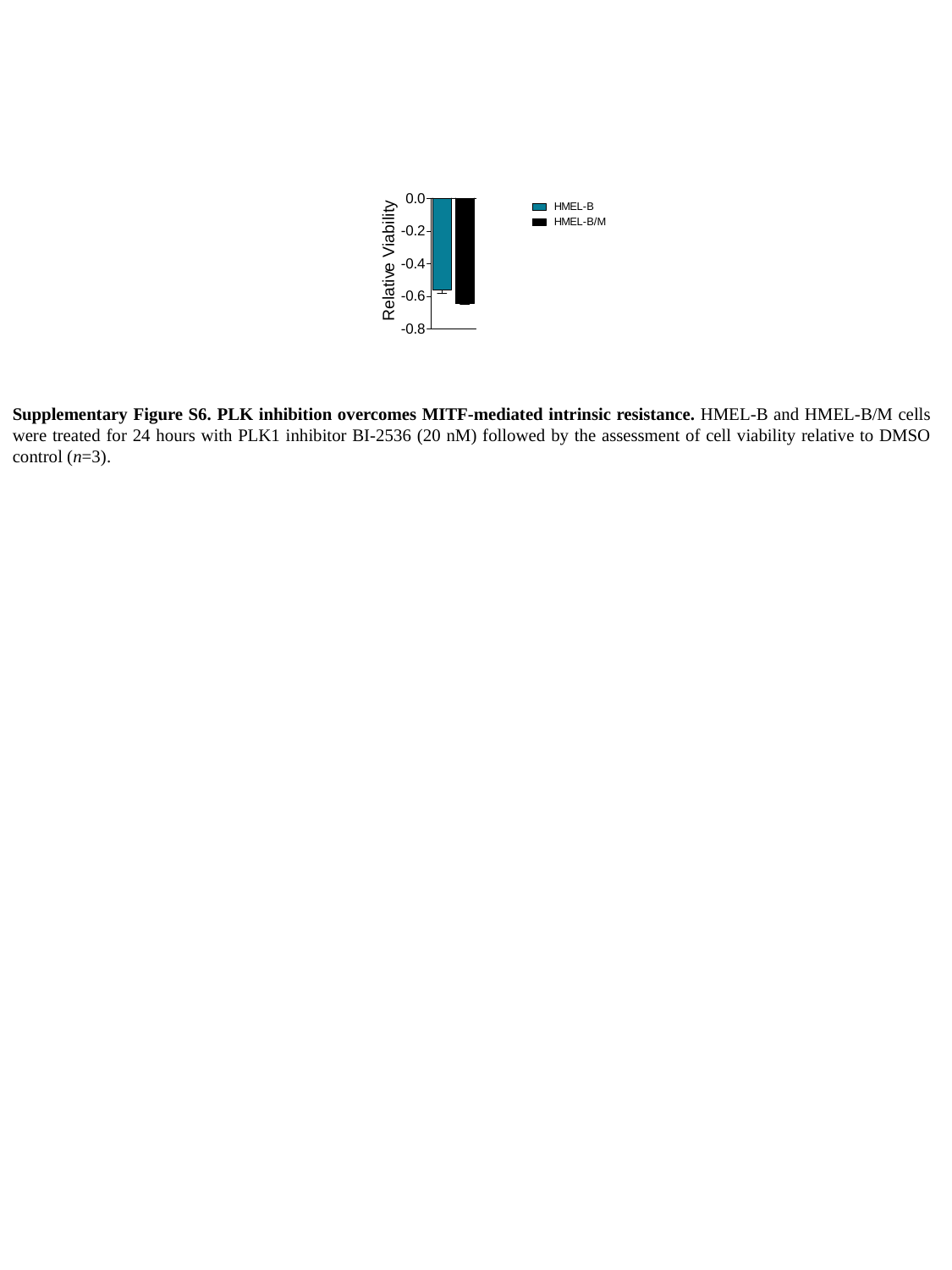

Supplementary Figure S6. PLK inhibition overcomes MITF-mediated intrinsic resistance. HMEL-B and HMEL-B/M cells were treated for 24 hours with PLK1 inhibitor BI-2536 (20 nM) followed by the assessment of cell viability relative to DMSO control (n=3).
